# Identifying Low pH Active and Lactate-Utilizing Taxa within Oral Microbiome Communities from Healthy Children Using Stable Isotope Probing Techniques

**DOI:** 10.1371/journal.pone.0032219

**Published:** 2012-03-05

**Authors:** Jeffrey S. McLean, Sarah J. Fansler, Paul D. Majors, Kathleen McAteer, Lisa Z. Allen, Mark E. Shirtliff, Renate Lux, Wenyuan Shi

**Affiliations:** 1 Microbial and Environmental Genomics, The J. Craig Venter Institute, San Diego, California, United States of America; 2 Pacific Northwest National Laboratory, Richland, Washington, United States of America; 3 Department of Microbial Pathogenesis, Dental School, University of Maryland, Baltimore, Maryland, United States of America; 4 School of Dentistry, University of California Los Angeles, Los Angeles, California, United States of America; 5 Washington State University Tri-Cities, Richland, Washington, United States of America; Cairo University, Egypt

## Abstract

**Background:**

Many human microbial infectious diseases including dental caries are polymicrobial in nature. How these complex multi-species communities evolve from a healthy to a diseased state is not well understood. Although many health- or disease-associated oral bacteria have been characterized *in vitro*, their physiology within the complex oral microbiome is difficult to determine with current approaches. In addition, about half of these species remain uncultivated to date with little known besides their 16S rRNA sequence. Lacking culture-based physiological analyses, the functional roles of uncultivated species will remain enigmatic despite their apparent disease correlation. To start addressing these knowledge gaps, we applied a combination of Magnetic Resonance Spectroscopy (MRS) with RNA and DNA based Stable Isotope Probing (SIP) to oral plaque communities from healthy children for *in vitro* temporal monitoring of metabolites and identification of metabolically active and inactive bacterial species.

**Methodology/Principal Findings:**

Supragingival plaque samples from caries-free children incubated with ^13^C-substrates under imposed healthy (buffered, pH 7) and diseased states (pH 5.5 and pH 4.5) produced lactate as the dominant organic acid from glucose metabolism. Rapid lactate utilization upon glucose depletion was observed under pH 7 conditions. SIP analyses revealed a number of genera containing cultured and uncultivated taxa with metabolic capabilities at pH 5.5. The diversity of active species decreased significantly at pH 4.5 and was dominated by *Lactobacillus* and *Propionibacterium* species, both of which have been previously found within carious lesions from children.

**Conclusions/Significance:**

Our approach allowed for identification of species that metabolize carbohydrates under different pH conditions and supports the importance of Lactobacilli and Propionibacterium in the development of childhood caries. Identification of species within healthy subjects that are active at low pH can lead to a better understanding of oral caries onset and generate appropriate targets for preventative measures in the early stages.

## Introduction

Oral biofilms represent an easily accessible model system for investigating inter-species interactions and functional relationships within multi-species communities [1], [2]. Despite their significant health-related relevance and many years of research [2], [3], [4], [5], oral biofilms remain enigmatic regarding critical interactions that occur at the community level. Most hypotheses that are still driving the bulk of dental research are based on cultivated isolates and single-species in animal models using germ-free environments. For dental caries, the most prevalent oral disease, these artificial experimental conditions led to the hypothesis that very few species contribute to disease initiation and progression. For example, *Streptococcus mutans* was identified early on as a major player in active caries and has been the primary subject of many caries research studies ever since. Other species that were classified as cariogenic based on their ability to ferment carbohydrates into acids similar to *S. mutans* include *S. sobrinus* and *Lactobacillus* spp [6]. However, an increasing number of culture-independent studies comparing the bacterial flora of healthy and diseased teeth suggest that these “classic” cariogenic species may be a small part of a more complex community involved with onset and progression of the disease. Studies on childhood caries in particular have found *Actinomyces*, *Fusobacterium*, *Porphyromonas*, *Selenomonas*, *Bacteriodetes*, *Haemophilus*, *Veillonella*, *Leptotrichia*, *Thiomonas*, *Streptococcus* and *Lactobacillus* in caries active lesions [7], [8], [9], [10], [11], [12]. Clearly, substantial clinical evidence is available to support the hypothesis that cariogenic activity of an oral biofilm is a complex polymicrobial disease whose etiology is based on the ability of the community to produce acid under decreasing pH conditions. The “ecological plaque hypothesis” developed by Marsh and colleagues [13] reflects this shift in our understanding of oral diseases by stating in essence that oral caries and periodontal diseases arise as a result of environmental perturbations to the habitat that cause shifts in the balance of the resident microbiota. Key features of this hypothesis are that (a) the selection of ‘pathogenic’ bacteria is directly coupled to changes in the environment and (b) diseases do not need to have a specific etiology; any species with relevant traits (i.e. acid production) can contribute to the disease process. Thus, physiological characteristics of newly discovered species could possibly predict their significance for disease.

For caries, environmental perturbations arise from the intermittent introduction of dietary sugars by the host that leads to cycling of pH with an immediate decrease after carbohydrate addition, followed by a slow steady rise in pH [14]. According to the ecological plaque hypothesis, if the pH remains below the “critical pH” for demineralization of 5.5 for extended time periods, a shift in the bacterial populations to more cariogenic organisms that are acid-producing (acidogenic) and acid-tolerant (aciduric) occurs [6], [15], [16]. These species then increase in numbers and worsen the diseased state since they thrive under acidic conditions and can outcompete other commensal species (i.e. those that raise the pH or antagonize cariogenic species). As this cycle continues, a predominance of cariogenic bacteria can emerge and lead to sustained demineralization of enamel and dentin. This presumed disease progression has been modeled *in vitro* through laboratory studies in chemostats using defined mixed communities [17], but the *in vivo* relevance remains to be confirmed.

Efforts of the Human Microbiome project (HMP) and recent advances in high-throughput genomics now allow for a comprehensive survey of bacterial species present in the oral cavity and how species composition varies among individuals [18], [19], [20], [21]. Through isolation and culture-independent methods, the curated Human Oral Microbiome Database (HOMD) contains 619 validated taxa with 1,178 total taxa identified, of which 24% are named, 8% are cultivated but unnamed, and 68% are uncultivated phlyotypes [20]. Although uncultivated representatives are found in diseased patients and therefore potentially linked to oral diseases, there is at present little understanding of their metabolic potential and ecological roles. In addition, most of what is known about the cultured oral species to date has been the result of isolation and pure culture studies of type strains, which may not reflect their actual physiology when competing in complex microbial communities.

SIP [22], [23] offers a tremendous potential to link function with species identity through the detection of metabolic networks that define microbial communities for *in vivo* as well as *in vitro* samples. Despite being a valuable tool for discovery of species function [24], [25], [26], [27], [28], SIP has been almost exclusively applied to environmental samples. In this study we expanded its powerful potential to oral microbiome samples and demonstrated that SIP can be used to identify the metabolically active species present within these complex multi-species communities under different pH conditions. Discovering novel and confirming known cariogenic species via their ability to function at low pH within healthy oral communities and ultimately relating these species to the onset and progression of disease can lead to better understanding of how communities shift from healthy to diseased states as well as develop improved biomarkers of the disease onset.

To link organic acid production and the specific bacterial species active in oral biofilms, we combined the nucleic acid based SIP with *in situ* non-invasive Magnetic Resonance Spectroscopy (MRS). The experimental procedure involved incubating supra-gingival plaque samples from healthy juvenile human subjects with isotopically labeled carbon sources (^13^C- glucose or ^13^C-lactate) in a defined minimal medium under various pH and buffering conditions. The temporal metabolite profiles of these live samples were monitored by ^1^H MRS. In addition, DNA and RNA SIP were performed on these samples and clone libraries were constructed of the heavy fractions representing the metabolically active species as well as the light fractions representing the overall sample diversity. Using this application, we demonstrated that this approach allows culture-independent identification of metabolically active species in complex oral plaque samples under varied environmental conditions relevant to dental caries.

## Results

### Metabolite Profiles under Neutral and Low pH Conditions

**Figure 1:**
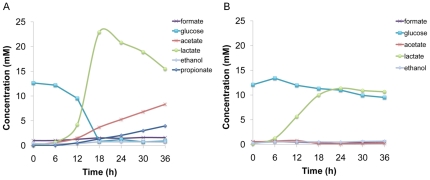
Temporal ^1^H MRS analyses of metabolites during glucose utilization in live plaque samples incubated with CDM media and ^13^C-glucose. (A) buffered CDM at an initial pH of 7 with 2 mM phosphate buffer and (B) unbuffered CDM at an initial pH of 5.5.

In order to gain insight into community metabolite profile progression, metabolic activities in plaque samples were monitored at 37°C within the NMR under the following experimental conditions: 1) buffered at pH 7 to reflect a non-cariogenic state and 2) low pH conditions with no buffering to simulate initial caries active conditions. Samples from healthy subjects were incubated with ^13^C-labeled glucose in CDM buffered to pH 7 or unbuffered with a starting pH of 5.5. Glucose utilization was rapid and complete in the buffered system (Figure 1A). Upon glucose depletion at 19–20 hrs, lactate utilization was evident accompanied by an increase in acetate and minor amounts of formate and ethanol. In contrast, the unbuffered sample adjusted to an initial pH of 5.5 (Figure 1B) was marked by inhibition of glucose fermentation with lactate as the major byproduct.

### RNA and DNA based Stable Isotope Probing

Isotopically labeled samples were subjected to either RNA or DNA extraction followed by density gradient centrifugation. Total RNA and DNA were obtained at 6 and 36 hrs, respectively. Density gradient centrifugation of the ^13^C-labeled samples resulted in distinct heavy and light peaks (Figure S1). The quality of the SIP procedure was confirmed by the absence of the peak representing the heavy fraction in the negative control containing non-isotopically labeled glucose. Further, a large discernable shift in the heavy fraction of treatment samples was indicative of active incorporation of labeled material by the microbial community.

#### RNA Stable Isotope Probing

**Figure 2:**
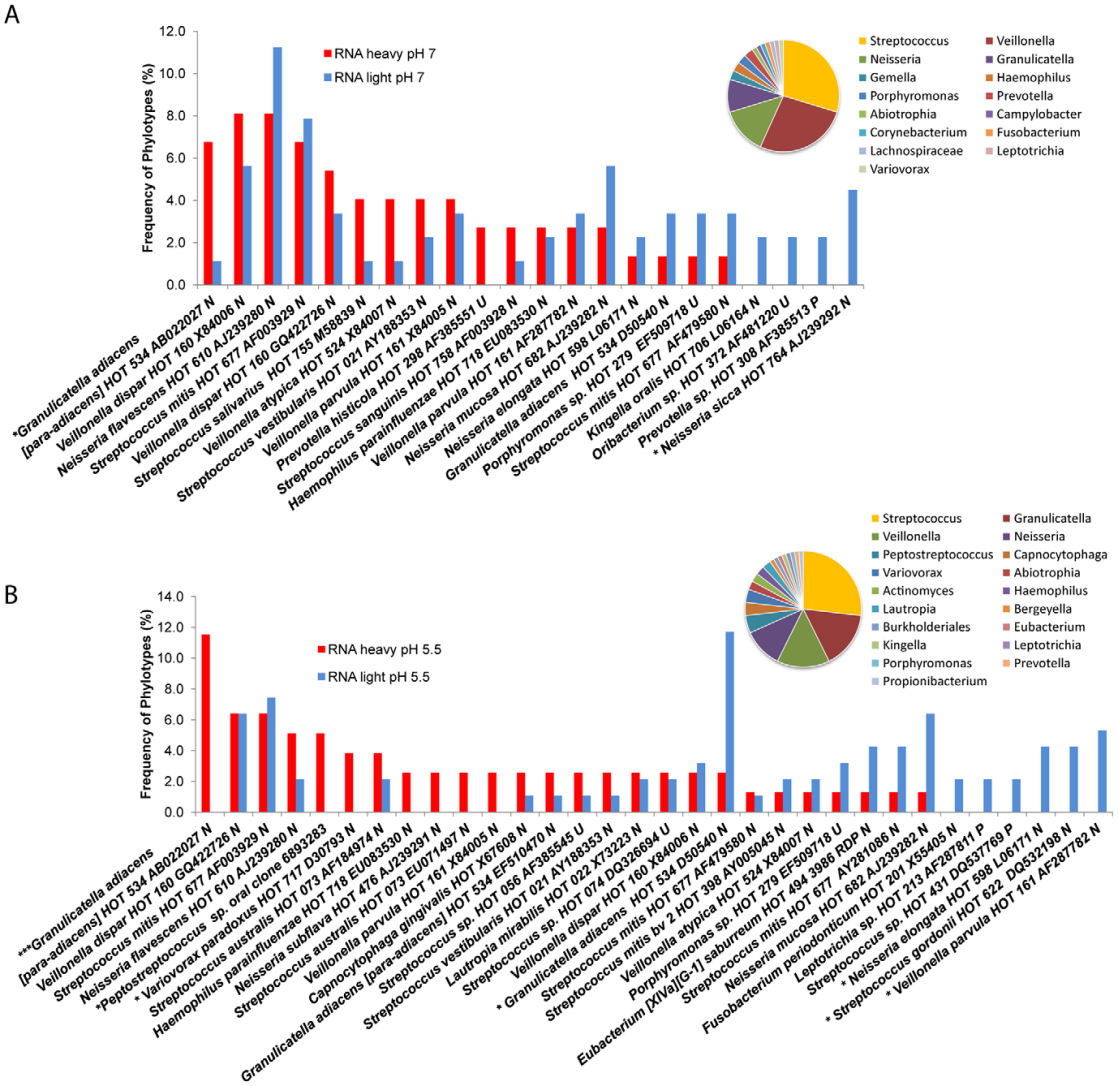
Comparative analyses of 16S rRNA sequences observed in the light and heavy fractions from samples incubated with ^13^C-glucose for 6 hrs. (**A**) buffered pH 7 conditions and (**B**) unbuffered pH 5.5 conditions. Data are reported for the dominant members that represent >2% of clones in the libraries. Taxa that differed significantly in detection frequencies are noted at *p<0.05, **p<0.01, ***p<0.001. The distribution of active genera in the heavy fractions is shown in the corresponding pie charts for each condition.

RNA-SIP was used to investigate the active species at 6 hrs of incubation when the glucose utilization was in progress and approximately 2 mM of lactate accumulation had occurred (Figure 1). Comparative analyses of 16S rRNA sequences observed in the light and heavy fractions from samples incubated with ^13^C Glucose for 6 hrs under buffered pH7 conditions and unbuffered pH5.5 conditions are shown in Figure 2. Data are reported for the dominant members that represent >2% of clones in the libraries. Species that differed significantly in detection frequencies between the heavy and light clone libraries are noted at *p<0.05, **p<0.01, ***p<0.001. The distribution of active genera represented by the heavy fractions is shown in the corresponding pie charts for each condition. The distribution of all the active genera observed under buffered pH 7 conditions (Figure 2A) revealed that *Streptoccocus*, *Veillonella*, *Neisseria*, and *Granulicatella* were abundant. Active species at pH of 5.5 under unbuffered conditions (Figure 2B) exhibited a similarly relative high diversity indicating that this pH was tolerable for many genera. The active genera under this lower pH included the same major genera found in the pH 7 samples. Species that appeared to increase their relative activity at pH 5.5 included *G. adiacens (para-adiacens)* HOT 534 Strain TKT1 (p<0.001), *Variovorax paradoxus* HOT and an uncultivated species *Peptostreptococcus sp*. oral clone 6893283 (p<0.05). A number of taxa that are currently designated as unnamed cultivated taxon such as *Streptococcus sp.* HOT 056, *Streptococcus sp.* HOT 074 and *Porphyromonas sp.* HOT 279 were also found to be active at this lower pH. Taxa that were present in the community and had a lowered apparent relative activity as indicated by their dominance in the light fraction (p<0.05) (p<0.05) included *G. adiacens* HOT 534 Strain GIFU12706, *N. elongate HOT* 598, *S. gordonii* HOT 622 *and V. parvula* HOT 161.

#### DNA Stable Isotope Probing

**Figure 3:**
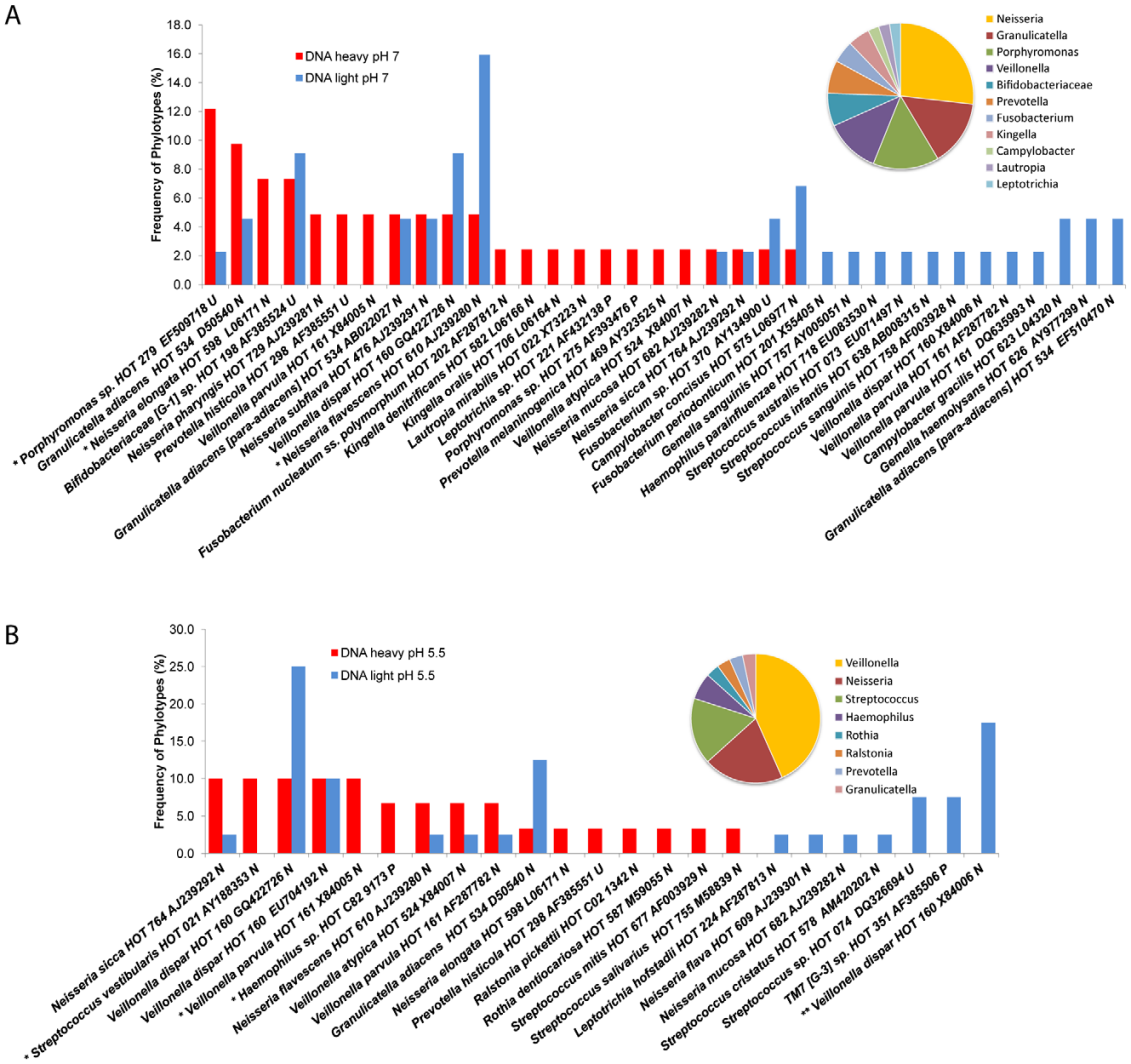
Comparative analyses of 16S rRNA sequences observed in the light and heavy fractions DNA SIP experiments incubated with ^13^C-glucose for 36 hrs. (**A**) under buffered pH 7 conditions, (**B**) unbuffered pH 5.5 conditions. Data are reported for the dominant members that represent >2% of clones in the libraries. Taxa that differed significantly in detection frequencies are noted at *p<0.05, **p<0.01, ***p<0.001. The distribution of active genera in the heavy fractions is shown in the corresponding pie charts for each condition.

The most abundant genera (>2% frequency) detected in the light and heavy DNA libraries (DNA-SIP) under buffered pH 7 conditions largely overlapped with the genera found in the RNA-SIP samples. In addition *Porphyromonas*, *Bifidobacteriaceae*, and *Veillonella* species were present, while streptococci were not abundant (Figure 3A). To further investigate which species are active and dividing at lower pH conditions that are relevant for sustained enamel demineralization, DNA-SIP was conducted under unbuffered conditions with an initial pH of 5.5. Under these experimental conditions (Figure 3B), *Veillonella*, *Neisseria*, and *Streptococcus* were again the dominant active genera. *S. vestibularis* HOT 021, *V. parvula* HOT 161, *Prevotella histicola* HOT 298. The uncultivated taxa *Haemophilus sp.* HOT C82 also exhibited activity (p<0.05) (Figure 3B). In contrast, *V. dispar* HOT160 which comprised 17% of the total clones (p<0.01) as well as *Streptococcus sp.* HOT 074 and uncultivated phylotype *TM7 [G-3] sp.* HOT 351 (representing each 7% of the total clones), were not active at the lower pH conditions.

Next, we sought to identify the species that can still thrive at the more extreme pH 4.5, a condition similar to a diseased-state which is hypothesized to drive a community shift to more aciduric species [17]. The community composition of this sample was different from the previous samples for DNA or RNA-SIP at pH 7 and pH 5.5. OTU-based analyses resulted in a lower number of observed OTUs and the relative loss of microbial diversity was evident in the diversity indices for this sample (Table 1). Comparing the Shannon diversity indices in a relative manner, there is a general trend indicating that a more acidic environment (lowering the pH from 7 to 4.5) resulted in a reduction in species richness. Due to the complexity of this system and the level of sequencing in this study, we recognize that this is a relative comparison, and that greater sequencing depth is necessary to more accurately determine species gain or loss.

**Table 1:** Comparison of phylotype coverage and diversity estimation of the 16S rRNA gene libraries at 3% dissimilarity.

Library	OTUs	#Seq	Shannon Index	95% C.I.	
				low	high
DNA Lactate Heavy	**17**	74	**2.06**	1.8	2.3
DNA Lactate Light	**22**	67	**2.51**	2.2	2.8
DNA Heavy pH 4.5	**13**	85	**1.42**	1.1	1.7
DNA Light pH 4.5	**18**	86	**1.47**	1.1	1.8
DNA Heavy pH 5.5	**18**	70	**2.00**	1.7	2.3
DNA Light pH 5.5	**10**	55	**1.74**	1.5	2.0
DNA Heavy pH 7	**19**	44	**2.70**	2.5	2.9
DNA Light pH 7	**17**	47	**2.52**	2.3	2.8
RNA Heavy pH 5.5	**31**	83	**2.99**	2.8	3.2
RNA Light pH 5.5	**24**	94	**2.67**	2.5	2.9
RNA Heavy pH 7	**22**	74	**2.56**	2.3	2.8
RNA Light pH 7	**31**	89	**2.86**	2.6	3.1

**Figure 4:**
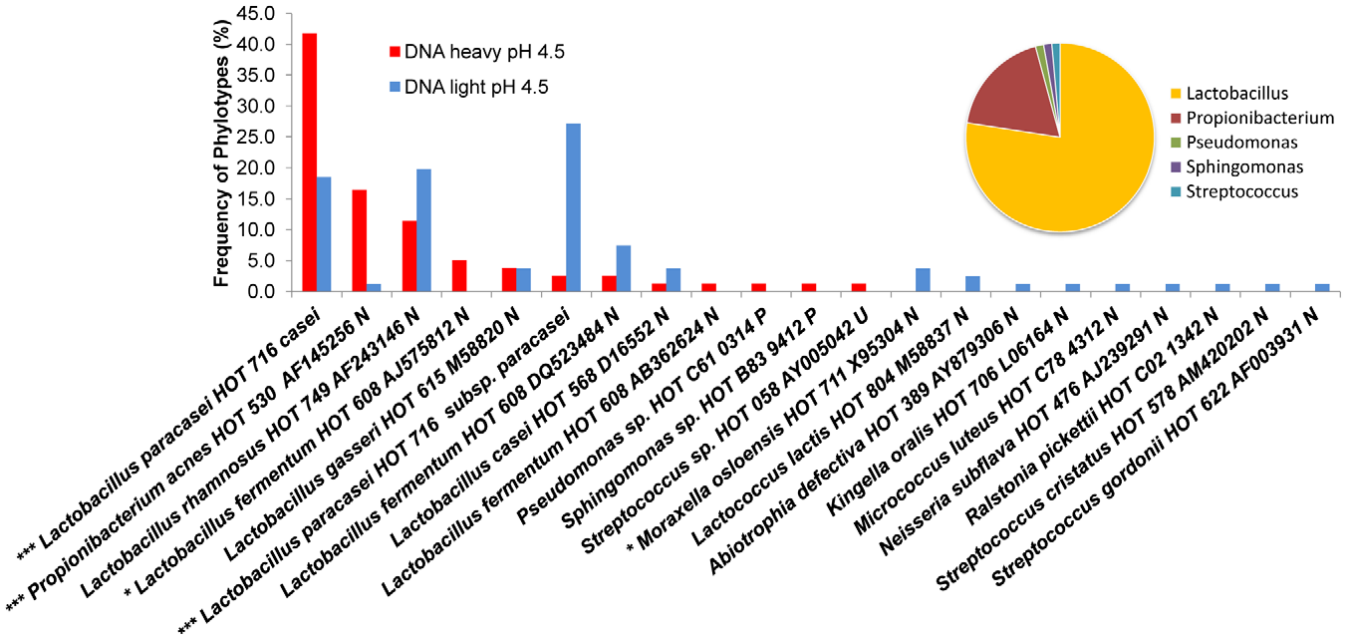
Comparative analyses of 16S rRNA sequences observed in the light and heavy fractions DNA SIP experiments incubated with ^13^C-glucose for 36 hrs at pH 4.5. Comparative analyses of 16S rRNA sequences observed in the light and heavy fractions DNA SIP experiment incubated with ^13^C-glucose at pH 4.5 for 36 hrs under unbuffered pH 4.5 conditions. Taxa that differed significantly in detection frequencies are noted at *p<0.05, **p<0.01, ***p<0.001. The distribution of active genera in the heavy fractions is shown in the corresponding pie charts for each condition.

Several members of the genus *Lactobacillus* represented 55% of the clones exhibiting activity at pH 4.5 (Figure 4), in addition to a single *Propionibacterium* species that comprised 13% of the total clones and was closely related to *P. acnes* HOT 530 Strain 63597 (sequence identity 99.2%). The same taxon was also observed at 1% frequency in the RNA-SIP libraries for the pH 5.5 unbuffered condition supporting its enhanced activity at low pH.

DNA-SIP results at pH 4.5 with ^13^C-glucose revealed significant differential growth within the genus *Lactobacillus*. In the heavy fractions, *L. fermentum* HOT 608 Strain AJ575812 represented 5% of the total clones with an increase over the number found in the light fractions (*p<0.05). A high percentage of 16S rRNA sequences fell within the *L. paracasei* HOT 716 HOMD taxon. This taxon contains 222 variable length sequences grouped at 98.5% and includes the closely related species *L. casei*, *L. paracasei* and *L. paracasei subspecies paracasei* [20]. Sequences related to HOT716 (72 sequences) were further analyzed using phylogenetics for sequence diversity within this goup (Figure S2). Two distinct clades were evident. The most prevalent containing 42% of sequences was phylogenetically placed with *L. casei* ATCC334 and this clade included primarily the heavy (active) fraction. In contrast, sequences that were placed with *L. paracasei subspecies paracasei Akira1* AY369076 represented 27% of the total sequences in this library and predominately contained the light (inactive) fraction. This indicates that although this particular strain was abundant in the sample it was apparently not as metabolically active under these conditions. For the purposes of this study, these sequences are designated *L. paracasei* Oral Taxon 716 *casei* and *L. paracasei* Oral Taxon 716 *subsp. paracasei* (Figure 4).

#### Linking microbial populations to lactate metabolism

**Figure 5:**
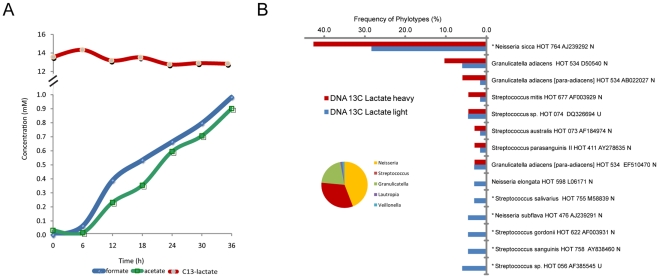
Live monitoring of ^13^C-lactate metabolism in plaque samples combined with DNA-SIP. (A) *In vivo* MRS metabolite profiles of active metabolism for the plaque community incubated with ^13^C-lactate buffered at pH 7. (B) Comparative plot of the major taxa (>2%) detected in the heavy (active) and light fractions. Taxa that differed significantly in detection frequencies are noted at *p<0.05, **p<0.01, ***p<0.001.

**Figure 6:**
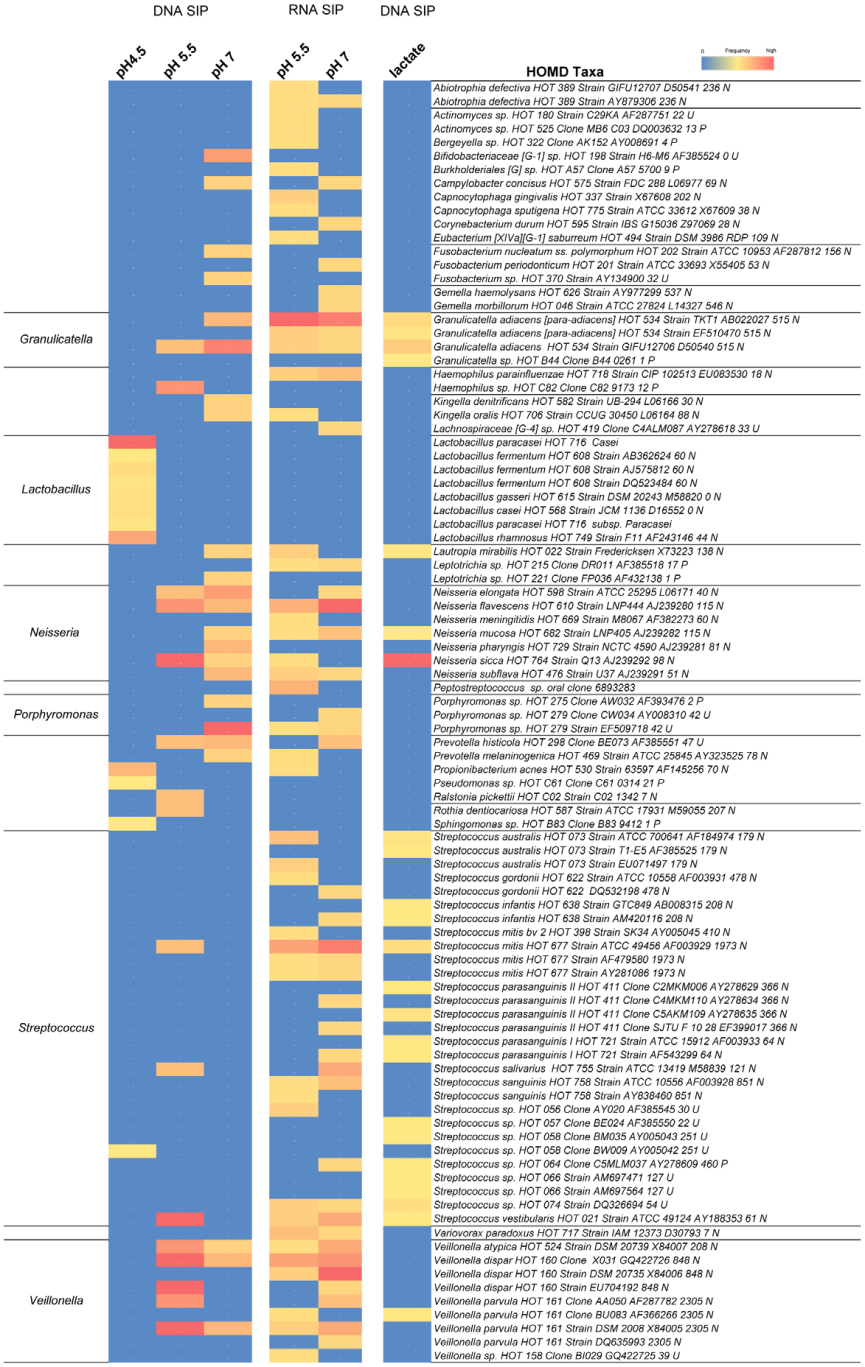
All taxa identified in the isotopically heavy fractions representing the active members for each condition. Heatmap of active members grouped by taxa in the ^13^C-DNA or RNA isotopically heavy fractions. Columns are mapped as the frequency of clones observed for each taxa within the respective clone library.

*In vivo* MRS experiments at pH 7 with ^13^C-labeled glucose as carbon source (Figure 1A) revealed a rapid rise in lactate concentrations followed by a decrease of lactate after glucose was exhausted. The utilization of lactate was accompanied by an increase in acetate and formate. This indicates that at least under buffered conditions, lactate is available for further utilization. In order to identify these lactate-metabolizing species, a sample was prepared according to the procedures described (materials and methods) and incubated with 14 mM of ^13^C-lactate without glucose in CDM buffered to pH 7 with phosphate buffer (Figure 5). In the absence of glucose only 1 mM of ^13^C-lactate was converted to formate and acetate. Sequencing of the respective clone libraries obtained for heavy and light fractions revealed that the most dominant genera in the heavy fraction and thus the metabolically active were *Neisseria*, *Streptococcus* and *Granulicatella* (Figure 6b) with *N. sicca* HOT 764 being the most dominant active taxon (p<0.01). *Veillonella* strains, which are known to be able to grow on lactate, were active under these conditions as expected. However, this genus comprised only 1.5% of the total clones in the heavy fraction.

#### Active Taxa

A heatmap display of all the active taxa observed across the experiments is shown in Figure 6. Several taxa were not only metabolically active but also able to divide at low pH as reflected by their representation in the pH 5.5 RNA-SIP (6 hr) and pH 5.5 DNA-SIP (36 hr) samples. These taxa include *G. adiacens* HOT 534, *N. flavescens* HOT 610, *N. sicca* HOT 764, *S. mitis* HOT 677, *S. vestibularis* HOT 021, *V. atypica* HOT 524, *V. dispar* HOT 160 and *V. parvula* HOT 161.

## Discussion

Our current understanding of microbial metabolism in the oral cavity is based almost entirely on single-species model studies. While this approach is suitable for the basic understanding of the metabolic capabilities of an organism, it cannot reflect behavior in a multispecies environment and account for the competitive or synergistic influence of other uncharacterized species present in these communities. This knowledge gap is a result of the prior inability to study metabolism directly in live oral biofilms and then linking this observed metabolism to the active species. In this study, this limitation was overcome by combining non-invasive and non-destructive MRS of live microbial communities with nucleic acid based SIP of species within mixed-species communities. The synergistic combination of SIP with traditional NMR on frozen and extracted samples was previously demonstrated for *in vitro* models for human colon [36] and intestinal communities [37]. NMR spectroscopy and spectroscopic imaging are non-invasive techniques capable of revealing the metabolic and mass transport processes in live prokaryotic cell suspensions, gel-immobilized cells [38], as well as live biofilms [39], [40]. Both temporal and depth-resolved metabolite concentrations within active oral biofilms of *S. mutans* have been shown previously [30]. The study presented here employs such an approach to understanding the dynamic metabolic interactions in an oral microbial community. Our working hypothesis for these experiments was that a low pH environment in a healthy plaque simulates conditions associated with oral caries progression and the corresponding microbial community shifts. Only those bacteria that can tolerate acid and continue to metabolize ^13^C-glucose (and the resulting byproducts) will have significant incorporation of the heavy isotope and be detected in the heavy labeled nucleic acid fractions. In particular, discovering cariogenic species (both established and unknown) that display the ability to function at low pH within healthy oral communities and relating these species to disease onset and progression can lead to the identification of better biomarkers for the disease.

### Community metabolite profiles

Demineralization, the prerequisite for cariogenesis and tooth decay exhibits a strong correlation with biofilm induced pH reduction, but a detailed understanding of the metabolic processes responsible for acid formation is lacking. The *in vivo* metabolite profiles of a supra-gingival oral community under “diseased” (low pH) and “healthy” (neutral pH) environmental conditions are unknown. An earlier NMR based study [41] analyzed extracts from plaque samples representing mixed microbial metabolism byproducts from the utilization of dietary sugars. Their results indicated that lactate, acetate, pyruvate, propionate, formate and n-butyrate are produced in abundance, with acetate and formate produced in higher concentrations than lactate [41]. To our knowledge, quantitative measurements of metabolite dynamics have not been conducted in a non-invasive manner on active oral biofilm communities. The metabolite profiles presented in this study allow a more detailed look at the temporal dynamics of the bacterial-derived acidic metabolites. The MRS results confirm that the conversion of glucose was complete at pH 7 but not at a starting pH of 5.5 under unbuffered conditions (Figure 1). The buffered pH 7 profiles clearly demonstrate the conversion of glucose to lactate and acetate followed by a shift in the metabolism toward lactate utilization when glucose became limiting. The main end products at 36 hrs for both conditions were lactate and acetate. Minor amounts of propionate, formate and ethanol were produced under buffered pH 7 conditions whereas propionate was not detectable under pH 5.5. The final pH at the end of the experiment was pH 6.02 and 5.03 for the buffered pH 7 and unbuffered pH 5.5 experiments respectively. Although only endpoint pH measurements were attainable for these in-magnet experiments, it is assumed that these plaque samples have a qualitatively similar “Stephan curve” [14] pH profile induced from glucose addition which is well documented response. Further technical advancements are needed to perform in-magnet pH profiles to correlate with the observed metabolite profiles.

### Low pH active Species

It is well documented that in oral plaque during a pulse of sugar, the production of acidic metabolites such as lactic acid causes a rapid decrease in pH [6], [14], followed by a slow steady rise in pH [14]. Sustained periods of low pH are thought to be associated with a shift in the oral microbial population to more aciduric and acidogenic species [5], [13], [15]. The species that can continue to metabolize at low pH in healthy plaque would therefore be considered important contributors to sustained production of acid. The results presented in this study demonstrated that RNA-SIP [42] and DNA-SIP [43] can be employed to reveal the species that are still able to thrive under increasingly low pH conditions. Very recently SIP methods have been used in conjunction with pyrosequencing of 16s rRNA to examine environmental samples, yielding short reads but higher coverage of less abundant species [44]. In our study, 16S rRNA clone libraries were generated from each of the gradient fractions containing either ^12^C (light) or ^13^C (heavy) nucleic acids (DNA or RNA). ^13^C-labeling of RNA is indicative of metabolic activity without necessitating growth, whereas ^13^C-labeling of DNA is generally thought to require cell doubling [45]. In this community, we were not only interested in the identity of the species that are active and incorporating isotopically heavy carbon (the “heavy” fraction) but also sought to identify species that were part of the community but not significantly active (the “light” fraction). To gain this information, a 16s rRNA clone library was generated for both the heavy and light fractions for all experimental conditions. Overall, the species present correspond to those seen with deep pyrosequencing studies conducted on healthy plaque samples from children with *Streptococcus*, *Granulicatella Veillonella*, *Neisseria*, *Haemophilus* and *Porphyromonas* being the dominant genera detected [46].

The DNA-SIP and RNA-SIP fractions from 36 hrs and 6 hrs of incubation, respectively, show that incorporation of ^13^C-labeled carbon from the isotopically labeled glucose and metabolic byproducts during the course of the incubation readily occurred (Figure S1). The high diversity of taxa and similarity to the light fraction observed in the heavy fraction (Table 1) under the simulated healthy condition (buffered pH 7) indicates that the medium used was not highly biased to certain groups. The results also support that the species able to be active in healthy plaque at pH 5.5 can be a diverse group of community members that are tolerant to this acidity level *in vivo*, likely due the periodic exposure to this low pH during the pH fluctuation after carbohydrate intake [14]. Both the RNA pH 5.5 and DNA pH 5.5 experiments identified *Neisseria*, *Granulicatella*, *Streptococcus* and *Veillonella* as being very metabolically active at this pH and likely acting collectively to lower the pH further from this point. HOMD *t*axa with the highest frequency found within these genera include *G. adiacens* HOT 534 Strain GIFU12706, *N. flavescens* HOT 610 Strain LNP444, *N. sicca* HOT 764 Strain Q13, *S. mitis* HOT 677 Strain ATCC 49456, *S. vestibularis* HOT 021 Strain ATCC 49124, *V. atypica* HOT 524 Strain DSM 20739, *V. dispar* HOT 160 Clone X031 and *V. parvula* HOT 161 Strain DSM 2008. *Streptococcus* and *Veillonella* are often in high abundance within severe early childhood caries (S-ECC) subjects [7], [8], [10], [12], [47]. In a recent study, *V. atypica* HOT 524 was correlated with S-ECC [48]. *G. adiacens* HOT 534 was also found in 41% of S-ECC subjects but also found in caries free subjects [48] although in this same study, *G. elegans* HOT 596 was highly correlated with *S-ECC. Neisseria* is a predominant member of oral communities from children [46] and has been found in relatively high numbers in S-ECC subjects [10]. *N. flavescens* HOT 610 was significantly correlated with S-ECC [48]. Although the subjects in our study were caries free, a number of low pH active taxa were identified that have been correlated with S-ECC subjects.

In contrast to the diversity of species able to function at an initial pH of 5.5, the diversity was dramatically reduced after 36 hrs during incubation at an initial pH of 4.5. The decrease in the number of OTU's and the loss of diversity (Table 1) was expected to some extent given the extreme pH of 4.5. At this very low pH, we observed species previously associated with caries-active subjects. Members of the genus *Lactobacillus* became dominant as well as a single *Propionibacterium* species. There was differential labeling within the *Lactobacillus* taxa (*L. paracasei* HOT 716). Taxa closely related to *L. casei* were highly active, whereas those closely related to *L. paracasei subsp. paracasei* were not. In addition, our results show that *L. rhamnosus, L .fermentum, and L. gasseri* were also active species at pH 4.5. *L. fermentum* , a known heterofermentative species, is frequently associated with the initiation of dental caries [49], [50] and found in association with nursing caries along with *L. gasseri* [51]. *L. gasseri*, *L. rhamnosus*, and a novel *Lactobacillus* phylotype were numerically the most dominant species within carious dentine samples [52]. There is also strong evidence that lactobacilli are associated with severe childhood caries. Members of these species have been isolated on acid pH 5 agar from S-ECC subjects [8] and were recently confirmed as dominant species in severe childhood caries by 16s rRNA analyses [10]. In addition, our SIP experiments suggest a functional difference between closely related sequences that form distinct groups with either *L. casei* and *L. paracasei subsp. paracasei.* Increased efforts to classify these species correctly in caries active subjects are clearly needed [53], [54], [55].

Members of the genus *Propionibacterium* are commonly detected in carious dentin of children and young adults [12] as well as found associated with caries in a number of molecular studies. In a recent analysis of the microbiota in dentinal caries, Munsun *et al.* [56] found that an as yet uncharacterized *Propionibacterium* taxon was the dominant *Propionibacterium* taxon present and one of only three taxa at that could be described at the species level to be found in every lesion. A recent study by Tanner *et al.* [8] also isolated *Propionibacterium* on acid pH 5 agar from both S-ECC subjects and caries free subjects. In our study, we found *P. acnes* HOT 530 Strain 63597 (98.6% sequence identity) was highly active at pH 4.5 increasing from 1% to 15% (p<0.0005) in the light and heavy fractions, respectively. Our results strongly suggest that the prevalence of *Propionibacterium* in carious lesions is related to their ability to function at low pH which facilitates its potential involvement in caries progression by sustaining this low pH. Gross *et al.* [10] recently found that *Propionibacterium* FMA5 was indeed highly associated with early childhood caries progression. Our study strongly agrees with the overall findings of their investigations into the species detected in clinical samples that range from early- to late-stage caries.

Discernable differences in the community members between caries-free and caries-active subjects have been observed. For example, Corby *et al.* [9] reported on the bacteria associated with caries and health in a subset of 204 twins aged 1.5 to 7 years old. They concluded that a strain of an *Actinomyces* species, *S. mutans*, and *Lactobacillus spp*. were more associated with disease. In contrast, bacterial species, including *S. parasanguinis*, *Abiotrophia defectiva*, *S. mitis*, *S. oralis*, and *S. sanguinis*, predominated in the indigenous bacterial flora of caries-free subjects [9]. As stated by Aas *et al.* [12] the findings of these studies and previous studies their own [57] demonstrated that there is a distinctive microbiota of the healthy oral cavity that is different from that associated with oral disease. In general, however, the polymicrobial nature of oral caries and the possible redundant and overlapping functions of many different species have confounded strict identification of the species involved in the disease initiation process. Our findings reveal that indeed species including currently uncultivated taxa are able to function at pH 5.5 besides those currently considered to be the main cariogenic organisms such as mutans group streptococci and lactobacilli. The results confirm that the contributions to initial acid production and low pH metabolism can result from collective community metabolism involving many diverse species and support the polymicrobial disease model. As the pH drops below pH 4.5 however, there is a pronounced shift in the species that are able to tolerate and continue to grow. A two stage shift has also been described in chemostat models with a defined mixed species consortium [17]. Bradshaw and Marsh [17] noted that a drop in pH to a value between pH 7 and 5.5 may allow the enrichment of potentially cariogenic species, whilst permitting species associated with health to remain relatively unaffected. They then stated that a further reduction in pH (<pH 4.5) may not only enhance the competitiveness of odontopathogens, but inhibit the growth and metabolism of non-caries-associated species. Our results indicate that within healthy plaque that is adjusted to pH 4.5 the species diversity decreases and that lactobacilli and *Propionibacterium* are actively dividing and becoming dominant members of the community which underscores their importance in the development of oral caries.

### Lactate Metabolism

An important part of the microbial processes related to the development and length of sustained acidic conditions *in vivo* is the production of lactate but also the conversion of lactate to other organic acids. Lactate utilization has also been linked to a rise in pH after the initial pH decrease [58], [59]. In our study, live temporal metabolite analyses indicated that lactate produced during glucose utilization was being converted to acetate and formate by unknown members of the community (Figure 5). Sandham and Kleinberg [59] had previously reported the utilization of lactate once glucose was exhausted at initially low glucose concentrations which are the same as our starting concentrations (13.9 mM). An earlier NMR based study which analyzed extracts from plaque samples, Silwood *et al.* [41] were concerned that previous studies of carious lesions have failed to detect and consider the contribution of formic acids to demineralization of tooth surfaces. The production of formic acid from lactate could contribute significantly to the decreased pH considering the larger dissociation constants compared to lactate (formic acid K_a_ = 1.77×10^−4^ mol/dm^3^; lactate K_a_ = 1.40×10^−4^mol/dm^3^) [41].

Given the questions surrounding the utilization of lactate and its unknown contribution to healthy or cariogenic conditions, we sought to investigate which microbial groups may be contributing to this observed lactate utilization with DNA-SIP employing isotopically labeled lactate (^13^C-lactate) as the sole carbon source. SIP results from this sample directly after metabolite measurements in the NMR showed oral bacteria capable of lactate utilization including both cultivated and uncultivated taxa. Representatives of the taxa found in the heavy DNA fractions (>2% frequency) included *N. sicca* HOT 764, *G. adiacens* HOT 534, *V. parvula* HOT 161 and a number of *Streptococ*cus taxa; *S. mitis* HOT 677, *S. australis* HOT 073, *S. parasanguinis* II HOT 411 and *Streptococcus* sp. HOT 074.

Using ^13^C-NMR, Leighton *et al.* [60] showed that a strain of *Neisseria* growing on lactate resulted in the excretion of significant amounts of acetate into the culture supernatant. Oral *Neisseria* isolated from dental plaque have also been shown to produce acetate, CO_2_ as well as some pyruvate [61]. *Streptococcus* species are also known to use this pathway [62], [63] and *S. mutans* AHT in particular exhibited lactate utilization and conversion to acetate under aerobic conditions and low glucose concentration [64] which resulted in an increase in pH. *S. sanguinis* and *S. oralis* were also reported to oxidize L-lactate with oxygen present. The SIP results presented here indicate that not all *Streptococcus* species were active (indicated by the presence in the light fraction only) and that there were no overlapping strains of the same taxa in both the heavy and light fractions. To our knowledge the genus *Granulicatella* has not been reported previously to be capable of lactate, oxidation. To support this observed metabolic trait indicated by SIP, genome analyses of sequenced representatives in the *Granulicatella* genera (*G. adiacens* ATCC 49175 and *G. elegans* ATCC 700633) confirmed the presence of L-lactate dehydrogenase gene as did searches against *S. sanguinis*, *S. oralis* and *N. flavescens* SK114.

Fermentation of lactate by *Veillonella* is well documented. Surprisingly, only 1% of *Veillonella*, consisting of *V. parvula* HOT 161, were detected in the active fraction despite their documented ability to grow on lactate *in vitro* indicating that the *in vitro* behavior of some species may not be entirely reflective of their metabolism in a mixed oral biofilm community. Although further work is certainly needed to dissect the species responsible for lactate metabolism under various oxygen and pH conditions, we were able to confirm the earlier hypotheses that *Streptococci* metabolize lactate within the context of a mixed oral community.

Currently, the human oral cavity is one of the targets for the HMP and is now being intensely studied by a number of groups. Hundreds of individual strains implicated in health and disease have been or are in the process of being sequenced (http://hmpdacc.org/). With the reported phylotypes reaching over 1,000 [20], this very diverse and dynamic community is being described in terms of the species present but little is known about the behavior of even the cultured strains *in vivo* and especially the currently uncultured representatives. DNA and RNA based SIP investigations were performed in conjunction with real-time metabolite profiling via *in vitro* MRS on live oral plaque samples to demonstrate the utility of the approach and to gain possible insights into the onset of oral caries disease by studying healthy subjects. Our results demonstrate that it can be a discovery tool to determine species contributing to cariogenic conditions (actively metabolizing species at low pH) and also help reveal the role currently uncultured species may have in oral disease progression. In particular, we can discover the cariogenic properties of cultured and uncultured species such as their acid tolerance (aciduric properties) and ability to compete for resources within the context of a mixed species community.

## Materials and Methods

### Sample Collection

Oral plaque samples derived from caries-free children were obtained from the University of Maryland Baltimore Campus under appropriate IRB approval. Pooled supra-gingival plaque samples were collected from the buccal, lingual and interproximal tooth surfaces in both the mandible and the maxilla with a sterile cotton swab. Each of the thirty samples that were harvested from individual patients as part of a blind clinical study contained approximately 200 µL of plaque scrapings and some saliva from healthy pediatric subjects. These plaque samples were pooled and subsequently frozen as separate aliquots at −80°C with 15% glycerol until use.

### Media

A chemically defined medium (CDM) modified from a previously published minimal simulated saliva medium for oral species [29] was employed for the metabolism studies since it had been shown to be compatible with MRS studies on *S. mutans* metabolism [30]. This CDM was composed as follows (in g/L): (NH_4_)_2_SO_4_ (0.8); NaCl (0.6); ascorbic acid (0.5); MgCl_2_.6H_2_O (0.16); CaCl_2_.2H_2_O (0.01); cysteine hydrochloride (0.3). To this, 20 mL/L of a vitamin mix was added. The composition of the vitamin mix (g/L) was biotin (0.002); folic acid (0.002); pyridoxine hydrochloride (0.01); riboflavin (0.005); thiamine (0.005); nicotinic acid (0.005); pantothenic acid (0.005); vitamin B-12 (0.0001); p-aminobenzoic acid (0.005); thioctic acid (0.005). ^13^C-labeled glucose and ^13^C-L-lactate (specifically labeled at the methyl carbon) were obtained from Cambridge Isotope Laboratories. Glucose (2.5 g/L) and lactate (1.5 g/L) was added immediately prior to use. For the samples which required buffering, KH_2_PO_4_, (3.0) and K_2_HPO_4_, (2.5) were used. For the unbuffered experiments, K_2_HPO_4_ was removed and KH_2_PO_4_ (5) was added to keep the total phosphate concentration standardized.

### Sample Incubation

Pooled plaque samples were thawed on ice and incubated at room temperature for 8 hrs in the dark in buffered CDM medium without a carbon source to exhaust residual carbon sources. The samples remained in the original clumped or aggregated form after cryopreservation. Immediately prior to the addition of fresh medium, the samples were washed once in CDM without glucose by centrifugation (5,000×g for 6 min). The samples were again pelleted and CDM medium containing ^13^C-labeled or ^12^C- carbon source was added to the tube without resuspending the pellet. Non-labeled glucose was used for the pH 7 buffered and pH 5.5 non-buffered MRS studies, SIP was performed on replicate samples. ^13^C-labeled glucose and lactate were used for the pH 4.5 and lactate MRS study respectively and SIP was performed immediately after the MRS measurements. Samples were incubated at 37°C in an anaerobic candlejar for 6 hrs (RNA based SIP) or 36 hrs (DNA based SIP), respectively. For the live NMR studies, after the final step of adding CDM with the carbon source, a total of 540 µL of sample including medium were promptly transferred to a 5 mm NMR tube. Deuterium oxide (60 µL) was added to provide a ^2^H NMR signal for magnetic field stabilization. The labeled substrate comprised the primary carbon source present in the medium.

### NMR Measurements

A live sample was inserted into a NMR spectrometer stabilized at 37°C and operating at a ^1^H NMR Larmor frequency of 599.64 MHz. The metabolic content of the sample was monitored at 15 min intervals for 36.5 hrs, using a water-suppressed ^1^H NMR Bloch decay (Varian *presat*) pulse sequence. Sixteen 13.5 sec repetitions were performed, using a 10 sec delay followed by a 1.5 sec presaturation interval then a 6.6 µsec rectangular RF pulse and a 2 sec acquisition window (38,422 complex points with a spectral width of 10 kHz). The spectra were referenced and calibrated using an external reference sample composed of 1 mM DSS in 10% D_2_O/H_2_O, which were collected both immediately before and after the time series.

Data were processed using VNMR analysis software (Varian, Palo Alto, CA) by applying 2 Hz of exponential line broadening followed by Fourier transformation. The individual spectra were phased and carefully baseline corrected, and the area of representative spectral lines were integrated (for formate, ascorbate, glycerol, cystine, cysteine, pyruvate methyl and both ^13^C satellite peaks, acetate methyl and both ^13^C satellite peaks and lactate methyl and both ^13^C satellite peaks). The integrated peak areas were corrected for T1 relaxation effects and spin count, and calibrated to yield molar concentrations. Spectral assignments were confirmed using Chenomx (Edmonton, Canada) NMR-analysis software.

### DNA Isolation and ^13^C-enriched DNA Isolation

DNA was isolated with DNeasy Blood and Tissue Kit (Qiagen) by following the protocol for the lysis of Gram^+^ bacteria. DNA was eluted in a final volume of 200 μL water. Labeled DNA was separated by centrifugation against a CsCl gradient. The entire DNA extracted from each sample was loaded into the gradient solution. Gradient formation was achieved by centrifugation at 265,000×g for 66 hrs in a Beckman VTi65 rotor (Beckman Coulter, Inc., Fullerton, CA). Fractions were collected (400 µL) and the DNA was isolated using a Ym-100 Microcon column (Millipore). Columns were washed four times with TE buffer and purified DNA was eluted into 50 μL volumes.

### RNA Isolation and ^13^C-enriched RNA Isolation

RNA was extracted by resuspending the cells in 1 mL RNA protect bacteria reagent (Qiagen), followed by addition of 200 μL Tris-EDTA (30 mM Tris, 1 mM EDTA, pH 8.0). This suspension was passed through a Qiashredder column (Qiagen) and Proteinase K (15 mg/mL) was added according to the manufacturer's protocol. The Lysate was then used for total RNA extraction with the RNeasy Mini kit (Qiagen) and the resulting RNA was subjected to RNase free DNase digestion (New England Biolabs) at 37°C for 10 min.

To separate RNA into ^12^C and ^13^C labeled fractions, a Cesium Trifluoroacetate (CsTFA) gradient was constructed using a CsTFA solution (2 g/mL) (Amersham Bioscience) and 100 µL formamide to an average density of 1.755–1.795 g/mL [31]. The CsTFA solution was loaded with 500 ng of total RNA. Gradient formation was achieved by centrifugation at 265,000×g for 46 hrs in a Beckman VTi65 rotor (Beckman Coulter, Inc., Fullerton, CA). Fractions were collected (400 µL) and RNA isolated using isopropanol precipitation. Random hexamers were used to generate cDNA from the recovered RNA in each fraction (Superscript III, Invitrogen).

### DNA Analysis

The cDNA or DNA from each fraction was used as template for PCR. Phusion polymerase (New England Biolab) was used to create blunt ended PCR products for cloning. Each PCR reaction consisted of: 10 µL 5× solution, 1 µL 10 mM dNTP, 0.5 µM of each primer (27F and 907R [32]), 0.5 µL phusion polymerase, 100 ng template and H_2_O to a final volume of 50 µL. Cycling parameters were: 98°C for 1 min, followed by 25 cycles at 98°C for 15 sec, 56°C for 15 sec, and 75°C for 30 sec, with a final extension at 75°C for 10 min. PCR products were purified using the Min-elute PCR purification kit (Qiagen) and eluted into 20 µL EB. Purified PCR products were then visualized on an Agilent Bioanalyzer 7500 DNA (Agilent Technologies) chip to ensure the correct band was amplified. PCR products were then cloned into pCRII-blunt vector (Invitrogen) and transformed into *Escherichia coli* strain Top10. Individual clones were picked and sequenced (Agencourt Biosciences).

### Sequencing and Phylogenetic Analysis of 16S rRNA Gene Libraries

A total of 12 clone libraries (96 clones each) of the 16S rRNA gene were constructed from each of the heavy labeled and light unlabeled gradient fractions. Sequences from the 27F and 907R amplification were manually inspected and assembled into contigs of an average length of 880 bp when possible with Vector NTi software. The sequences were then analyzed for the presence of chimeras by using the CHIMERA_CHECK ver. 2.7 program of the Ribosomal Database Project (RDP)-II release 8.1 resulting in 868 total reads. The total number of sequences used in the analyses for each library is listed in Table 1. OTU based analyses were performed using MOTHUR (version1.18.0) (http://schloss.micro.umass.edu/mothur/Main_Page) [33] and the diversity indices calculated at evolutionary distance of 0.03. Sequences were submitted to the Human Oral Microbiome Database (www.HOMD.org) website for BLAST analysis [20], [34]. The top hits against the extended database (ver 1.1) were used with a cutoff at 97% identity. Each BLAST result from the HOMD database returns a strain or clone sequence from within a grouped Human Oral Taxon (HOT) [20]. In some cases, phylogentic approaches and additional sequences from the RDP database were used to further identify the closest representatives. Sequences were aligned using MUSCLE (Edgar, 2004) and maximum-likelihood trees constructed using PhyML (Guindon, 2003) with the GTR substitution model. In this study, the number of clones observed for each taxon between the light and heavy fractions was taken as an indication of their relative activity increase or decrease during the incubation period with *p*-values estimated using the two standard population proportions test for library comparisons [35].

### 16S rRNA Gene Sequence Accession Numbers

Accession numbers of the HOMD taxa are provided on figures. Unique sequences from clone libraries that did not meet the HOMD BLAST cutoff criteria will be deposited in NCBI prior to publication.

## Supporting Information

Figure S1 **Gradient fraction analysis by density.** Nucleic acid concentrations across density gradients from A) RNA-SIP and B) DNA-SIP analyses of the oral plaque samples incubated at without glucose (control) and with ^13^C-labeled glucose at pH 7 and pH 5.5.
(TIF)

Figure S2 **Evolutionary relationships of taxa found in the DNA of the heavy fraction under pH 4.5 incubation.** Sequences related to HOMD taxa *Lactobaccillus paracasei* HOT 716 were used in this analyses to further investigate the sequence diversity within this group. The relationship to the nearest neighbor (100% ID) sequence from the RDP database are shown (denoted by *).
(TIF)
