# Supplementary figures and images for: Identifying Low pH Active and Lactate-Utilizing Taxa within Oral Microbiome Communities from Healthy Children Using Stable Isotope Probing Techniques
Source: PLoS One. 2012 Mar 5;7(3):e32219. doi: 10.1371/journal.pone.0032219 (PMC3293899; doi:10.1371/journal.pone.0032219)

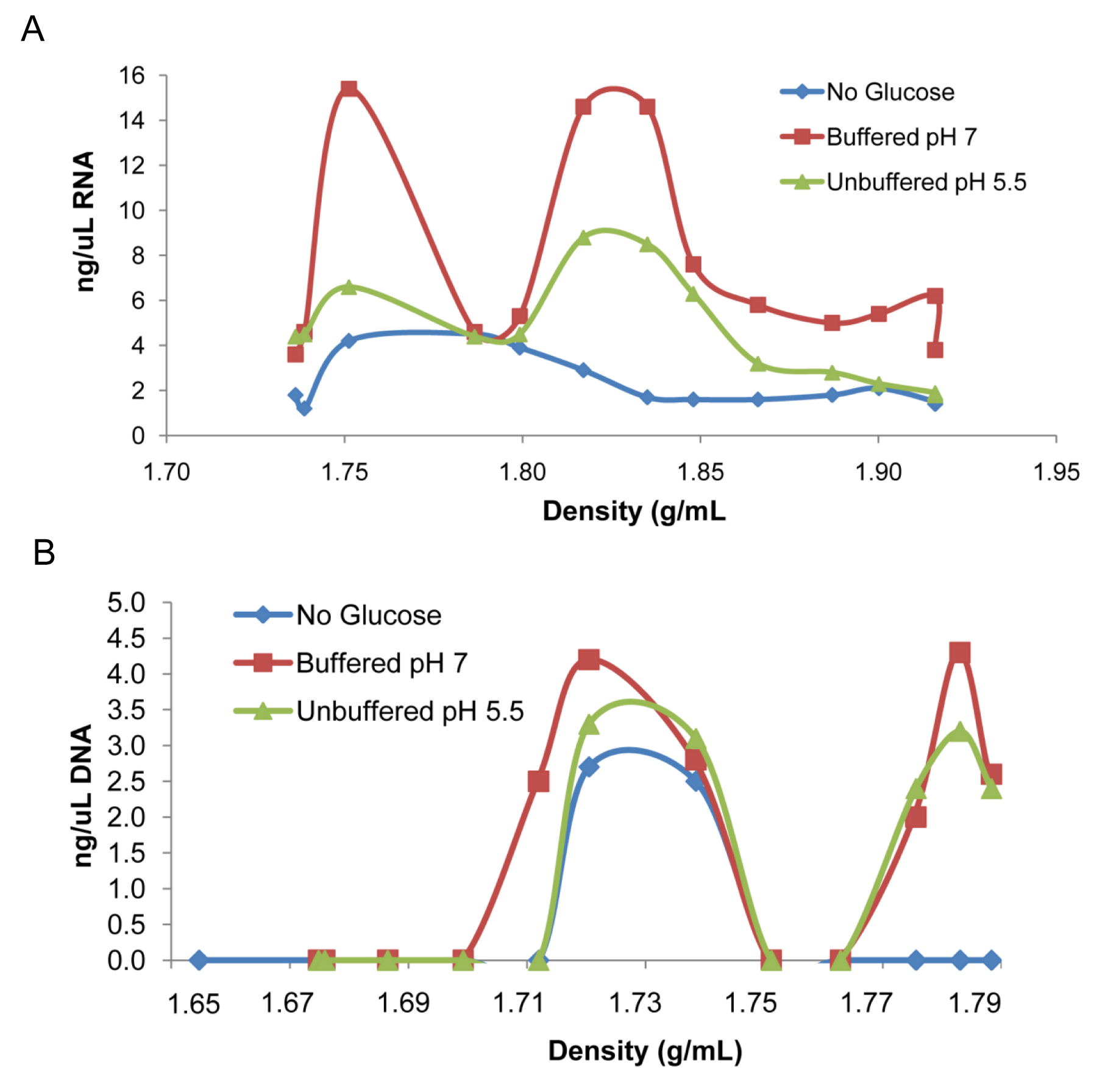

Supplement: Figure S1 — Gradient fraction analysis by density. Nucleic acid concentrations across density gradients from A) RNA-SIP and B) DNA-SIP analyses of the oral plaque samples incubated at without glucose (control) and with 13C-labeled glucose at pH 7 and pH 5.5. (TIF) [file pone.0032219.s001.tif]

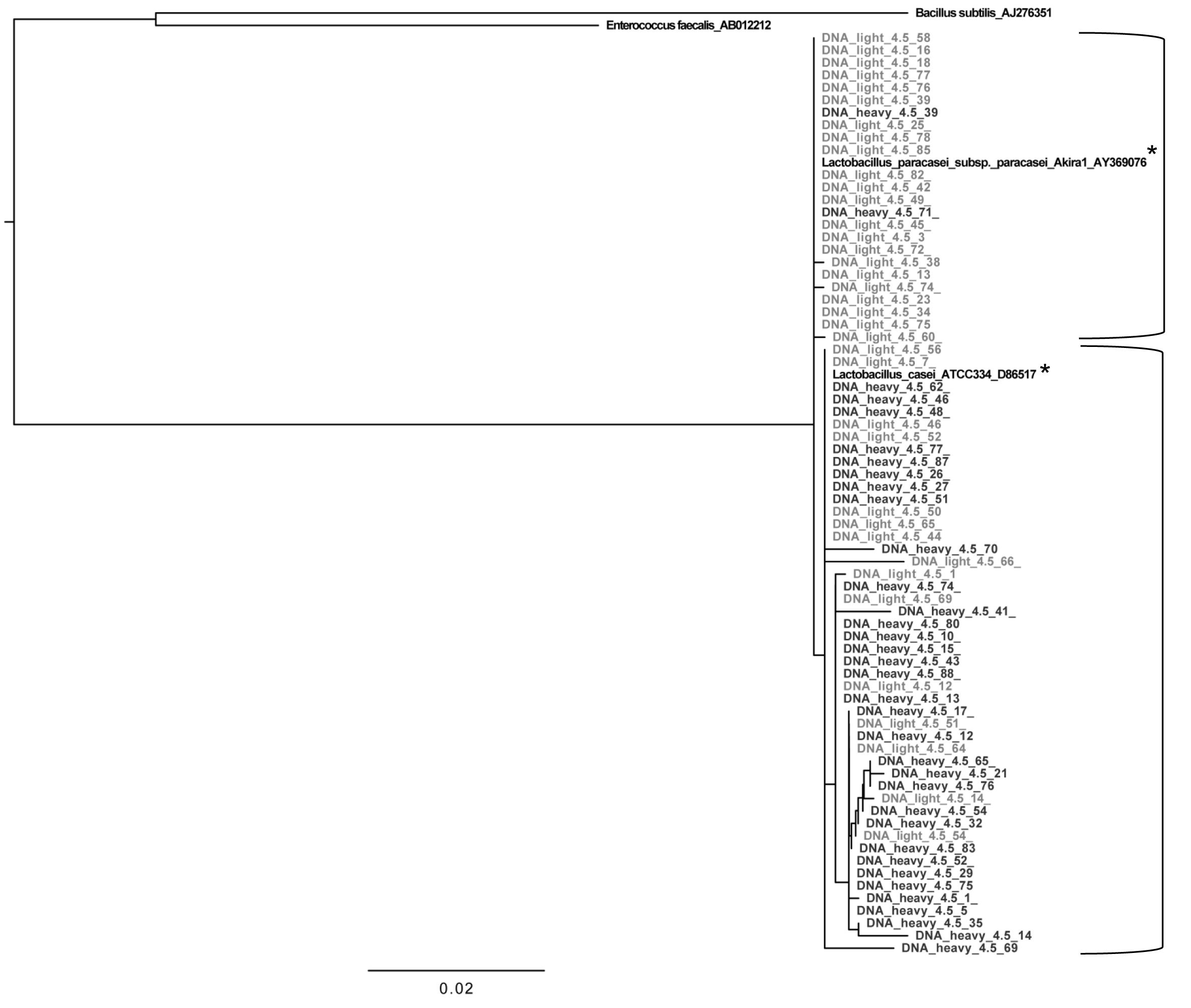

Supplement: Figure S2 — Evolutionary relationships of taxa found in the DNA of the heavy fraction under pH 4.5 incubation. Sequences related to HOMD taxa Lactobaccillus paracasei HOT 716 were used in this analyses to further investigate the sequence diversity within this group. The relationship to the nearest neighbor (100% ID) sequence from the RDP database are shown (denoted by *). (TIF) [file pone.0032219.s002.tif]
